# Pulsed‐Field Ablation in Management of Ventricular Tachycardia: A Systematic Review of Case Reports and Clinical Outcomes

**DOI:** 10.1002/clc.70018

**Published:** 2024-10-01

**Authors:** Amir Askarinejad, Erfan Kohansal, Amirreza Sabahizadeh, Hamed Hesami, Sara Adimi, Majid Haghjoo

**Affiliations:** ^1^ Rajaie Cardiovascular Medical and Research Institue Iran University of Medical Sciences Tehran Iran; ^2^ School of Medicine Shiraz University of Medical Sciences Shiraz Iran; ^3^ Cardiovascular Epidemiology Research Center Rajaie Cardiovascular Medical and Research Institute Tehran Iran; ^4^ Cardiac Electrophysiology Research Center, Rajaie Cardiovascular Medical and Research Institute Iran University of Medical Sciences Tehran Iran; ^5^ Department of Cardiac Electrophysiology, Rajaie Cardiovascular Medical and Research Institute Iran University of Medical Sciences Tehran Iran

**Keywords:** catheter ablation, pulsed‐field ablation, ventricular arrhythmia, ventricular tachycardia

## Abstract

**Background:**

Pulsed‐field ablation (PFA) is a cutting‐edge technique that employs non‐thermal energy to cause cell death by inducing irreversible electroporation of cell membranes. This systematic review evaluates the PFA effectiveness as a potential alternative to radiofrequency and cryo‐ablation for treating ventricular tachycardia.

**Methods:**

PubMed, Embase, Scopus, and Web of Science were systematically searched using keywords related to ventricular tachycardia and pulsed‐field ablation. Eligible Studies evaluating this therapeutic approach for ventricular tachycardia were included in the final analysis.

**Results:**

We included six studies (five case reports and one case series) in our systematic review. Eight (88.8%) of procedures were successful with 100% long‐term efficacy. No procedural complications or ventricular tachycardia (VT) recurrence were observed in the cases.

**Conclusion:**

The absence of complications, high effectiveness, and long‐term success rate make PFAs a good VT treatment option. However, PFA safety and efficacy studies for VT treatment are scarce. Thus, larger investigations on this topic are urgently needed.

## 1 Introduction

Ventricular tachycardia (VT) is a life‐threatening arrhythmia that accounts for a significant portion of the estimated 300,000 sudden cardiac deaths (SCDs) occurring annually in the United States alone [1, 2, 3]. VT is an abnormal heart rhythm that arises from tissue below the penetrating AV bundle and causes a rapid ventricular rate above 100 beats per minute. VT may occur in hearts with normal or pathological structures and can have either stable or unstable hemodynamics [4]. Symptoms of VT include syncope, cardiogenic shock, ventricular fibrillation, electrical storm, cardiac arrest, and SCD [5].

Current management of VT involves evaluating the risk of SCD, considering the use of implantable cardioverter‐defibrillators (ICDs) when appropriate and managing VT by treating underlying myocardial disease using antiarrhythmic medications, performing catheter ablation, and exploring other developing treatments [4].

Catheter ablation aims to eliminate the arrhythmogenic substrate or interrupt re‐entry circuits by delivering energy to destroy targeted heart tissue. Traditional methods include radiofrequency ablation (RFA), which uses heat, and cryoablation, which uses extreme cold [6]. Despite recent improvements in catheter ablation procedures, the results remain less than ideal, reflecting a high rate of relapse over the long term [7]. Furthermore, recent research has demonstrated that vascular complications are the most common cause of problems associated with VT ablation. Uncommon complications of the procedure include acute hemodynamic decompensation, cardio‐embolic events, valvular injury, aortic dissection, arrhythmias caused by ablation, and damage to the conduction system, coronary arteries, and surrounding tissue due to ablation [8].

Pulsed‐field ablation (PFA) is an innovative technique that uses non‐thermal energy to induce irreversible electroporation in cardio‐myocytes, creating targeted ablation lesions while sparing surrounding tissues [9]. Preclinical studies have demonstrated that PFA can create precise, controlled lesions with minimal collateral damage. These studies suggest that PFA could offer significant benefits over traditional ablation methods, such as radiofrequency and cryoablation [10, 11].

PFA's characteristics could be advantageous in VT ablation, including its capacity to generate extensive and uniform lesions while minimizing damage to nearby tissues, preventing scar contraction, and reducing the likelihood of coagulum formation. Also, Improved application speed (in seconds) can enhance stability and reduce procedural times [12, 13].

Despite promising preclinical findings, PFA for VT ablation in humans is still in its early stages. To address this knowledge gap and guide future research, we conducted a systematic review of available case reports and series on PFA for VT ablation in humans. Our aim was to assess acute procedural success rates, complications, and short‐term recurrence rates, providing an overview of the current state of this emerging technique and identifying areas for further investigation.

## 2 Materials and Methods

The protocol for this systematic review was previously registered at PROSPERO with an identification number CRD42023491662. This manuscript was prepared and reported according to the PRISMA 2020 statement [14].

### 2.1 Search Strategy

A thorough search was undertaken to find the potentially eligible articles in four electronic databases (PubMed, Web of Science, Scopus, and Embase) using relevant keywords with no specific restriction on the time frame, language, or article types since October 31, 2023. The detailed search strategy is presented in Table S1. The search results were then merged into one file using Endnote software version 21, which was used to find and remove duplicate records. The remaining records were then uploaded to the Rayyan web application for the initial screening of titles and abstracts [15].

### 2.2 Study Selection

Two reviewers (A.S. and H.H.) carried out the screening process using the Rayyan web app. Any studies evaluating the PFA in management of VT were included. Studies evaluating other arrhythmia treatment with this therapeutic approach were excluded. Reviews and editorials were also excluded. The records were labeled as either “Excluded” or “Maybe” during the screening based on the pre‐specified eligibility criteria. Records labeled as “Maybe” were then entered into the final stage of screening, with full texts being scrutinized for possible inclusion. Supplementary sources used to find additional records included bibliographies of the eligible articles and also previous meta‐analyses.

### 2.3 Data Extraction

E.K. and A.A. designed a data extraction form. These reviewers independently extracted data from all studies that met the eligibility criteria and resolved any disagreements through consensus. The subsequent information was extracted: the name of the first author, the year of publication, study design, country, reported cases gender, age, and underlying disease, medications, implanted device, previous ablations, VT morphology and origin, utilized mapping devices, utilized PFA catheter, and procedure description.

### 2.4 Risk of Bias and Quality Assessment

A.A. and E.K. assessed the quality of the studies using the JBI's critical appraisal tools for case reports [16] and case series [17]. A third reviewer (M.H.) was involved in cases of inconsistencies.

## 3 Results

**Figure 1:**
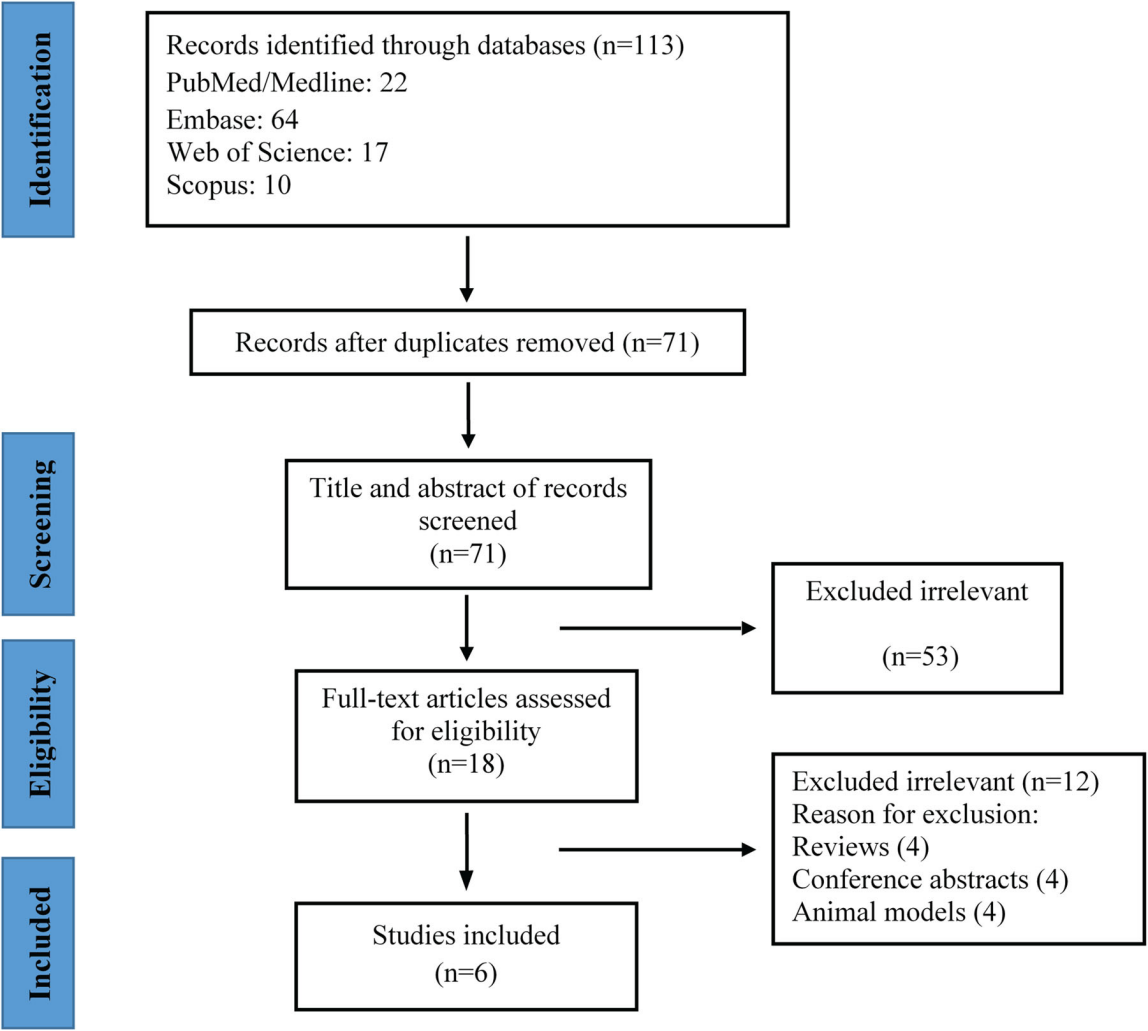
Flowchart of study selection for inclusion in the systematic review.

The study selection flow diagram is illustrated in Figure 1. A total of 113 papers were identified using systematic database searches, including PubMed, Embase, the Web of Science, and Scopus. After removing duplicate records, a total of 71 articles underwent further screening. Fifty‐three records were excluded based on an assessment of their title and abstract, as their topic of study or findings were considered unrelated to our research. We conducted an in‐depth assessment of 18 papers by examining their full‐text content. Six studies with a total population of eight individuals were ultimately included [18, 19, 20, 21, 22, 23].

### 3.1 Study and Patient Characteristics

A total of eight reported cases in six articles published in 2023, consisting of five case reports [18, 19, 21, 22, 23] and one case series [20], were included for systematic review (Table 1). These reports originated from various European countries, including Portugal, Germany, Spain, the United Kingdom, and the Netherlands. Study participants were predominately male (87.5%), with a mean age of 65.75 years (33–83 years). Underlying cardiac pathology included ischemic cardiomyopathy (two reports), nonischemic dilated cardiomyopathy, Ebstein's anomaly, arrhythmogenic cardiomyopathy, LV aneurysm, non‐obstructive coronary artery disease with preserved EF, and a prior myocardial infarction. Six patients were mentioned to have implanted cardiac devices and were on antiarrhythmic medications due to recurrent VT episodes (Table 2).

**Table 1:** Characteristics of included studies.

Reference	Country	Study design	Title
Adragao et al. [18]	Portugal	Case report	Pulsed‐field ablation vs radiofrequency ablation for ventricular tachycardia: First in‐human case of histologic lesion analysis
Krause et al. [19]	Germany	Case report	Flowerpower: pulsed‐field ablation of ventricular tachycardia in a patient with Epstein's anomaly
Lozano‐Granero et al. [20]	Spain	Case series	Case series of ventricular tachycardia ablation with pulsed‐field ablation
Martin et al. [21]	UK	Case report	First worldwide use of pulsed‐field ablation for ventricular tachycardia ablation via a retrograde approach
Ouss et al. [22]	Netherland	Case report	First in human pulsed field ablation to treat scar‐related ventricular tachycardia in ischemic heart disease: a case report
Weyand et al. [23]	Germany	Case report	First in human focal pulsed field ablation to treat an epicardial VT focus with an endocardial approach in nonischemic cardiomyopathy

Abbreviation: VT, ventricular tachycardi.

**Table 2:** Baseline clinical characteristics.

Reference	Gender	Age	Underlying disease	Medications	Device	Previous ablations/type	Type of previous ablations
Adragao et al. [18]	Male	60	Ischemic cardiomyopathy, LVEF < 20%, heart transplant waiting list	B‐blockers Amiodarone Mexiletine	CRT‐D	Three endocardial VT ablations for electrical storms refractory to medications	Endocardial
Krause et al. [19]	Male	33	Ebstein's anomaly of the tricuspid valve	Amiodarone	ICD	RFA of the sustained monomorphic VT	Endocardial
Lozano‐Granero et al. [20]	Male	83	Non‐obstructive coronary artery disease, preserved LVEF	—	—	—	—
	Female	83	Large LV aneurysm	Amiodarone Mexiletine	ICD	—	—
	Male	69	Arrhythmogenic cardiomyopathy with biventricular involvement	Sotalol	ICD	—	—
Martin et al. [21]	Male	68	Anterior myocardial infarction Post‐infarction ventricular tachycardia (VT)	—	—	Two VT ablation procedures, 4 and 3 years earlier	Both endocardial and epicardial
Ouss et al. [22]	Male	69	Ischemic cardiomyopathy, LVEF:30%	Amiodarone Bisoprolol	CRT‐D	Two VT ablation procedures, with the most recent one performed 6 months prior	Endocardial
Weyand et al. [23]	Male	61	Nonischemic dilated cardiomyopathy	B‐blockers Amiodarone	ICD	—	—

Abbreviations: CRT‐D, cardiac resynchronization therapy with a pacemaker and an ICD; ICD, implantable cardioverter‐defibrillators; LV, Left ventricle; LVEF, left ventricular ejection fraction; VT, ventricular tachycardia.

The morphologies of VT were mentioned in five cases, which were monomorphic for all of them, consisting of the right bundle branch block pattern (two cases) and the left bundle branch block pattern (two cases) with the superior axis. The cycle lengths of VTs ranged from 285 to 580 ms. VT origins were located in various parts of the ventricle, mostly in the septal region. Table 2 summarizes the underlying disease, medications, and previous ablations in the included studies.

### 3.2 Procedural Details and Outcomes

A variety of cardiac mapping and ablation delivery systems were utilized. Electroanatomic mapping systems (CARTO 3, Orion) paired with multi‐electrode mapping catheters (PENTARAY, OCTARAY) were used to guide and target ablation lesions in three studies [18, 20, 23]. Dedicated pulsed‐field ablation generators, catheters, and sheaths manufactured by Farapulse (FARASTAR, FARADRIVE, FARAWAVE) were used in five studies [18, 19, 20, 21, 22], with one study using the CENTAURI pulsed field system by Galaxy Medical paired with a THERMOCOOL SMARTTOUCHTM ablation catheter [23].

Pulsed‐field ablation was delivered either focally (one case) or in overlapping flower, radial, or basket‐type lesion sets using between seven and 56 overlapping individual ablation applications. Applications were generated using biphasic waveforms, pulse widths under 1 ms, voltages between 2000 and 4000 V, getting 50–100 pulses per application, and target ablation indices of ≥ 400. Table 3 demonstrates the procedural details in all cases of the final included studies.

**Table 3:** Reported cases of pulse field ablation procedural characteristics.

Reference	VT characteristics			Utilized mapping devices		Ablation type	Utilized PFA catheter and procedure description		
	Origin	Morphology	TCL (ms)	System	Catheter		PFA catheter	PFA parameters	Application technique
Adragao et al. [18]	Septal region	Left bundle branch block‐like morphology with and superior axis	580	CARTO 3	OCTARAY	PFA, RF	FARAWAVE	2000 V, 18 applications	Flowerlike
Krause et al. [19]	Anterior part of the atrialized RV	Sustained monomorphic	285	—	—	PFA	FARAWAVE	35 applications	Flower
Lozano‐Granero et al. [20]	Case 1: septal region Case 2: LV aneurysm Case 3: basal inferolateral RV free wall	Case 1: left bundle branch block–like morphology and superior axis Case 2: right bundle branch block morphology and superior axis Case 3: Electrical storm and multiple ICD shocks caused by incessant VT	Case 1: 580 Case 2: 520	CARTO 3	OCTARAY	PFA	FARAWAVE	2000 V, 9 applications	Basket
Martin et al. [21]	An area of scar posteriorly under the aortic valve, partially intramural	—	—	—	—	PFA	FARAWAVE	2000 V, 56 applications	Bipolar
Ouss et al. [22]	—	Distal anteroseptum AMI scar‐related VT	—	—	—	PFA	FARAWAVE	2000 V, 7 applications	Flower‐like
Weyand et al. [23]	Septal region	Right bundle branch block pattern with north‐west axis	319 ms	—	PENTARAY	PFA	THERMOCOOL SMARTTOUCH	—	—

Abbreviations: ICD, implantable cardioverter‐defibrillators; TCL, tachycardia cycle length; VT, ventricular tachycardia.

Details on procedural challenges and limitations were scarce across these early reports. However, Adragao and colleagues [18] did note difficulties in achieving adequate catheter contact and stability for pulsed‐field ablation delivery to portions of the left ventricle. They cited design factors of the mapping and ablation technologies used, including insufficient curve diameter of the sheath and non‐steerable catheter shaft. As a result, ablation to the anterior wall in their patient could not be performed as intended.

Regarding outcomes, PFA resulted in the successful elimination of VT in all cases except for one case in the study of Lozano‐Granero et al. [20] which had an epicardial origin in the right ventricle. In that case, despite initial VT termination with endocardial PFA delivery, VT remained inducible and required subsequent epicardial radiofrequency ablation to achieve non‐inducibility.

No procedural complications were found in the eight included cases. Interestingly, no VT recurrences were observed in all cases after follow‐up evaluations. Table 4 summarizes the procedure outcomes, VT recurrences, and complications in the final included studies.

**Table 4:** Procedural outcomes and recurrences in the studies.

Reference	Sample size	Successful procedure^a^ outcome (%)	VT recurrence	Procedural complications
Adragao et al. [18]	1	1 (100%)	The patient had no arrhythmic recurrence for 48 h following the ablation procedure.	0 (0%)
Krause et al. [19]	1	1 (100%)	No further episodes of VT were documented, neither on electrocardiogram (ECG) and Holter monitoring nor on ICD telemetry, and no further ICD discharges occurred since then during a 3‐month follow‐up.	0 (0%)
Lozano‐Granero et al. [20]	3	2 (66.6%)	Case 1: no recurrences have been documented during a 6‐month follow‐up. Case 2: no recurrences have been documented during a 5‐month follow‐up. Case 3: no recurrences have been documented during a 4‐month follow‐up.	0 (0%)
Martin et al. [21]	1	1 (100%)	The patient has subsequently remained free of VT during 2 months follow‐up.	0 (0%)
Ouss et al. [22]	1	1 (100%)	At 6 months follow‐up, the patient was free of VT.	0 (0%)
Weyand et al. [23]	1	1 (100%)	no recurrences have been documented during a 6‐month follow‐up.	0 (0%)

Abbreviations: ICD, implantable cardioverter‐defibrillators; VT, ventricular itachycardia.

a Successful procedure was defined as the inability to induce VT after the procedure.

### 3.3 Risk of Bias Assessment in the Studies

Quality appraisal was conducted for the five included case reports [18, 19, 21, 22, 23] using the JBI checklist for case reports [16] and for the case series [20] using the JBI checklist for case series [17].

Among case reports, three studies [19, 21, 22] received a perfect score (8/8 score), and the remaining two studies by Adragao et al. and Weyand et al. received 7 and 6 scores consecutively [18, 23]. The case reports were generally of good quality and low risk of bias, with clear descriptions of patient demographics, clinical conditions, interventions, and outcomes. Key lessons learned were summarized in all case reports (Table S2). However, Adragao et al. lost points for not clearly describing longer term post‐intervention outcomes since the patient underwent transplantation and for not discussing potential adverse events [18]. Similarly, Weyand et al. did not provide a detailed timeline of the patient history leading up to pulsed‐field ablation and also did not comment on complications or unintended negative effects [23].

The case series study by Lozano‐Granero et al. [20] received an 8/10 score, indicating good overall quality but some domains that were not applicable. The lack of statistical analysis was appropriate, given the small sample size.

## 4 Discussion

Our systematic review reveals that PFA demonstrates a high procedural success rate of 87.5% with no reported procedural complications across the included studies. This promising success rate underscores the potential of PFA as a viable alternative to traditional ablation methods. However, it is essential to acknowledge the limitations of the current studies, including small sample sizes and short follow‐up durations.

Preclinical studies have shown that PFA can create precise, controlled lesions with minimal collateral damage, which is particularly advantageous in VT ablation. These findings align with our review, where PFA exhibited excellent efficacy and safety profiles. Furthermore, our findings suggest that PFA's ability to minimize damage to surrounding tissues makes it a particularly promising technique for VT ablation. The success rate of 87.5% indicates that a significant majority of patients experienced successful ablation procedures with no immediate complications. This is noteworthy when compared to traditional ablation techniques, which often have higher complication rates and less precision in lesion formation. However, it is important to consider that the success rate is based on a limited number of cases and short‐term follow‐up. Larger studies with longer follow‐up periods are needed to validate these findings and to better understand the long‐term efficacy and safety of PFA.

The most important question is about the safety and efficacy of pulsed‐field ablation for the treatment of cardiac arrhythmias. Complications that can arise during radiofrequency or cryo‐ablation procedures for VT include vascular complications (most common), acute hemodynamic decompensation, cardio‐embolic events, and complications related to catheter manipulation such as valvular injury, aortic dissection, cardiac implantable electronic device lead dislodgement, and pro‐arrhythmia associated with ablation [8]. Considerably, 4%–8.5% of patients undergoing VT catheter ablation experience vascular complications, including pseudo‐aneurysm, retroperitoneal hemorrhage, arteriovenous fistula formation, transient limb ischemia, arterial dissection, and hematomas [24, 25, 26, 27].

Pulsed‐field ablation induces nonthermal lesions in cardiac tissue within milliseconds by means of irreversible electroporation [28]. When cardiac cells go through exposure to strong electric field gradients, their cell membranes become more permeable, resulting in cell death without significant protein denaturation or damage to tissue structure. Additional cellular types, such as those found in the esophagus and nerves, exhibit greater resilience towards such alterations [29]. This contrasts with traditional thermal ablation techniques, such as radiofrequency or cryoablation, which often cause collateral damage to surrounding structures [30].

In line with a low rate of complications (0.8%–2.5%) in recent trials of using PFA for managing atrial fibrillation [31, 32], none of the patients in our included studies had procedural complications. This presents a favorable opportunity for conducting large‐scale studies to assess the safety of PFA in the treatment of VT.

These early studies lacked sufficient information regarding the specific procedural obstacles and limits. Nevertheless, Adragao and colleagues [18] acknowledged the challenges encountered in maintaining sufficient catheter contact and stability when delivering pulsed‐field ablation to specific areas of the left ventricle. The design factors of the mapping and ablation technologies employed were cited, which included the sheath having an insufficient curve diameter and the catheter shaft being non‐steerable. Consequently, the anticipated ablation of the anterior wall could not be carried out in the patient. This issue highlights current restrictions in maneuverability and consistent tissue apposition for existing pulsed‐field ablation systems that will need to be optimized in next‐generation technologies before widespread adoption. Additional unforeseen challenges may emerge as use expands to more complex cases. However, as initial feasibility reports, limitations were likely underreported even in the successful cases.

In seven (87.5%) cases out of eight cases in the included studies, procedural success was achieved. In the investigation conducted by Lozano‐Granero et al. [20], despite the initial termination of VT via endocardial PFA administration, the VT persisted and necessitated further treatment with epicardial radiofrequency ablation to achieve non‐inducibility. This exception highlights potential limitations of the current depth of PFA lesion penetration, particularly in patients with non‐endocardial or thicker myocardial VT substrates. Additional studies are warranted to define optimal PFA techniques and catheter configurations for more consistent disruption of complex ventricular arrhythmia circuits.

Interestingly, none of the patients in the studies had VT recurrences after the follow‐up sessions. Analysis of multicenter registry data, along with extensive long‐term monitoring, indicates that 70% of the patients remain free from any recurrent VT, accompanied by a general decrease in the overall burden of VT [33, 34]. However, the pooled long‐term success rate of 100% based on our study has some limitations because of the low sample size and short follow‐up duration in one case; further large randomized studies are warranted to evaluate the long‐term outcome of PFA for VT management in comparison with conventional ablation procedures.

There are limitations to our study. The primary limitation is the small sample size of the included patients, as the papers that were included consisted of case reports and case series. Furthermore, we were unable to conduct any quantitative synthesis due to the design of the studies included. Additionally, there is a potential for reporting bias, including publication bias, selective reporting within studies, and language bias. Case reports and series may inherently highlight successful cases, potentially underreporting complications or failures. To mitigate these biases, we included a comprehensive search strategy and aimed to extract all relevant data from the included studies. Despite these efforts, future research should include larger studies with more standardized reporting to enhance the reliability and generalizability of the findings.

## 5 Conclusions

A high rate of success along with absence of complications make the PFA an optimal method for VT catheter ablation. Nevertheless, there is a scarcity of studies assessing the safety and effectiveness of PFA for the management of VT. Therefore, there is an urgent need for larger studies to be conducted on this particular subject.

## Declaration of Generative AI and AI‐Assisted Technologies in the Writing Process

During the preparation of this work, the authors used Claude 2.1 to English language fluency editing and ensure native quality writing. Claude was consulted regarding grammar, word choice, sentence structure, and overall clarity of expression. After using this service, the authors reviewed and edited the content as needed and take full responsibility for the content of the publication.

## Author Contributions

Majid Haghjoo and Amir Askarinejad designed the systematic question and study. Amir Askarinejad performed a systematic search. Amirreza Sabahizadeh and Hamed Hesami screened and selected the studies. Amir Askarinejad, Erfan Kohansal, and Amirreza Sabahizadeh drafted the manuscript. Amir Askarinejad and Sara Adimi revised the manuscript. All authors read and approved the final version of the manuscript.

## Conflicts of Interest

The authors declare no conflicts of interest.

## Supporting information

Supporting information.

Supporting information.

## Data Availability

The data sets used (extracted data from included studies) and analyzed during the current study are available from the corresponding author upon reasonable request.
