# Supplementary material for: Pulsed‐Field Ablation in Management of Ventricular Tachycardia: A Systematic Review of Case Reports and Clinical Outcomes
Source: Clin Cardiol. 2024 Oct 1;47(10):e70018. doi: 10.1002/clc.70018 (PMC11442986; doi:10.1002/clc.70018)
Supplement: Supplementary file 1 — Supporting information. [file CLC-47-e70018-s002.docx]

**Table S1**. Search syntaxes.

| **PubMed** | ("pulsed field ablation" [all] OR “pulsed field” [all] ) AND (“Ventricular Tachycardias” [all] OR “Ventricular Tachyarrhythmias”[all] OR (“Tachyarrhythmia” [all] AND “Ventricular” [all]) OR “Ventricular Tachyarrhythmia”[all] OR “Ventricular Tachycardia”[all] OR “Nonsustained Ventricular Tachycardia” [all] OR “Nonsustained Ventricular Tachycardias” [all] OR (“Tachycardia”[all] AND “Nonsustained Ventricular”[all]) OR (“Ventricular Tachycardia”[all] AND “Nonsustained”[all]) OR “Paroxysmal Supraventricular Tachycardia”[all] OR “Paroxysmal Supraventricular Tachycardias”[all] OR (“Supraventricular Tachycardia”[all] AND “Paroxysmal”[all]) OR (“Tachycardia” [all] AND “Paroxysmal Supraventricular”[all]) OR “Idiopathic Ventricular Tachycardia”[all] OR “Idiopathic Ventricular Tachycardias”[all] OR (“Tachycardia”[all] AND “Idiopathic Ventricular”[all]) OR (“Ventricular Tachycardia”[all] AND “Idiopathic”)) |
| --- | --- |
| **Web of Science** | ((ALL= (“pulsed field ablation”) OR ALL= (“pulsed field”))  AND(ALL= (“Ventricular Tachycardias”) OR ALL= (“Ventricular Tachyarrhythmias”) OR ALL= (“Tachyarrhythmia” AND “Ventricular” ) OR ALL= (“Ventricular Tachyarrhythmia”) OR ALL= (“Ventricular Tachycardia”) OR ALL= (“Nonsustained Ventricular Tachycardia”) OR ALL= (“Nonsustained Ventricular Tachycardias”) OR ALL= (“Tachycardia” AND “Nonsustained Ventricular”) OR ALL= (“Ventricular Tachycardia” AND “Nonsustained”) OR ALL= (“Paroxysmal Supraventricular Tachycardia”) OR ALL= (“Paroxysmal Supraventricular Tachycardias”) OR ALL= (“Supraventricular Tachycardia” AND “Paroxysmal”) OR ALL= (“Tachycardia” AND “Paroxysmal Supraventricular”) OR ALL= (“Idiopathic Ventricular Tachycardia”) OR ALL= (“Idiopathic Ventricular Tachycardias”) OR ALL= (“Tachycardia” AND “Idiopathic Ventricular”) OR ALL= (“Ventricular Tachycardia” AND “Idiopathic”))) |
| **Embase** | (((‘pulsed field ablation’) OR (‘pulsed field’))  AND  ( (‘Ventricular Tachycardias’) OR (‘Ventricular Tachyarrhythmias’) OR (‘Tachyarrhythmia’ AND ‘Ventricular’ ) OR (‘Ventricular Tachyarrhythmia’) OR (‘Ventricular Tachycardia’) OR (‘Nonsustained Ventricular Tachycardia’) OR (‘Nonsustained Ventricular Tachycardias’) OR (‘Tachycardia’ AND ‘Nonsustained Ventricular’) OR (‘Ventricular Tachycardia’ AND ‘Nonsustained’) OR (‘Paroxysmal Supraventricular Tachycardia’) OR (‘Paroxysmal Supraventricular Tachycardias’) OR (‘Supraventricular Tachycardia’ AND ‘Paroxysmal’) OR (‘Tachycardia’ AND ‘Paroxysmal Supraventricular’) OR (‘Idiopathic Ventricular Tachycardia’) OR (‘Idiopathic Ventricular Tachycardias’) OR (‘Tachycardia’ AND ‘Idiopathic Ventricular’) OR (‘Ventricular Tachycardia’ AND ‘Idiopathic’))) |
| **Scopus** | ((ALL (“pulsed field ablation”) OR ALL (“pulsed field”))  AND (ALL (“Ventricular Tachycardias”) OR ALL (“Ventricular Tachyarrhythmias”) OR ALL (“Tachyarrhythmia” AND “Ventricular” ) OR ALL (“Ventricular Tachyarrhythmia”) OR ALL (“Ventricular Tachycardia”) OR ALL (“Nonsustained Ventricular Tachycardia”) OR ALL (“Nonsustained Ventricular Tachycardias”) OR ALL (“Tachycardia” AND “Nonsustained Ventricular”) OR ALL (“Ventricular Tachycardia” AND “Nonsustained”) OR ALL (“Paroxysmal Supraventricular Tachycardia”) OR ALL (“Paroxysmal Supraventricular Tachycardias”) OR ALL (“Supraventricular Tachycardia” AND “Paroxysmal”) OR ALL (“Tachycardia” AND “Paroxysmal Supraventricular”) OR ALL (“Idiopathic Ventricular Tachycardia”) OR ALL (“Idiopathic Ventricular Tachycardias”) OR ALL (“Tachycardia” AND “Idiopathic Ventricular”) OR ALL (“Ventricular Tachycardia” AND “Idiopathic”))) |
