# Supplementary material for: Pulsed‐Field Ablation in Management of Ventricular Tachycardia: A Systematic Review of Case Reports and Clinical Outcomes
Source: Clin Cardiol. 2024 Oct 1;47(10):e70018. doi: 10.1002/clc.70018 (PMC11442986; doi:10.1002/clc.70018)
Supplement: Supplementary file 2 — Supporting information. [file CLC-47-e70018-s001.docx]

**Table S2.** Risk of bias assessment in the final included studies.

| **JBI checklist for case report** | **Adragao, 2023** | **Krause, 2023** | **Martin, 2023** | **Ouss, 2023** | **Weyand, 2023** | **JBI checklist for case series** | **Lozano-Granero, 2023** |
| --- | --- | --- | --- | --- | --- | --- | --- |
| **Were patient’s demographic characteristics clearly described?** | Yes | Yes | Yes | Yes | Yes | **Were there clear criteria for inclusion in the case series?** | Yes |
| **Was the patient’s history clearly described and presented as a timeline?** | Yes | Yes | Yes | Yes | No | **Was the condition measured in a standard, reliable way for all participants included in the case series?** | Yes |
| **Was the current clinical condition of the patient on presentation clearly described?** | Yes | Yes | Yes | Yes | Yes | **Were valid methods used for identification of the condition for all participants included in the case series?** | Yes |
| **Were diagnostic tests or assessment methods and the results clearly described?** | Yes | Yes | Yes | Yes | Yes | **Did the case series have consecutive inclusion of participants?** | Yes |
| **Was the intervention(s) or treatment procedure(s) clearly described?** | Yes | Yes | Yes | Yes | Yes | **Did the case series have complete inclusion of participants?** | Yes |
| **Was the post-intervention clinical condition clearly described?** | Yes | Yes | Yes | Yes | Yes | **Was there clear reporting of the demographics of the participants in the study?** | Yes |
| **Were adverse events (harms) or unanticipated events identified and described?** | No | Yes | Yes | Yes | No | **Was there clear reporting of clinical information of the participants?** | Yes |
| **Does the case report provide takeaway lessons?** | Yes | Yes | Yes | Yes | Yes | **Were the outcomes or follow up results of cases clearly reported?** | Yes |
|  | | | | | | **Was there clear reporting of the presenting site(s)/clinic(s) demographic information?** | N/A |
|  |  |  |  |  |  | **Was statistical analysis appropriate?** | N/A |

N/A: not applicable, U/C: unclear
